# Face Recognition and Visual Search Strategies in Autism Spectrum Disorders: Amending and Extending a Recent Review by Weigelt et al.

**DOI:** 10.1371/journal.pone.0134439

**Published:** 2015-08-07

**Authors:** Julia Tang, Marita Falkmer, Chiara Horlin, Tele Tan, Sharmila Vaz, Torbjörn Falkmer

**Affiliations:** 1 School of Occupational Therapy and Social Work, Curtin University, Perth, Western Australia, Australia; 2 School of Education and Communication, CHILD programme, Institution of Disability Research Jönköping University, Jönköping, Sweden; 3 Department of Mechanical Engineering, Curtin University, Perth, Western Australia, Australia; 4 Rehabilitation Medicine, Department of Medicine and Health Sciences (IMH), Faculty of Health Sciences, Linköping University & Pain and Rehabilitation Centre, UHL, County Council, Linköping, Sweden; 5 Cooperative Research Centre for Living with Autism Spectrum Disorders (Autism CRC), Long Pocket, Brisbane, Queensland, Australia; University of Tuebingen Medical School, GERMANY

## Abstract

The purpose of this review was to build upon a recent review by Weigelt et al. which examined visual search strategies and face identification between individuals with autism spectrum disorders (ASD) and typically developing peers. Seven databases, CINAHL Plus, EMBASE, ERIC, Medline, Proquest, PsychInfo and PubMed were used to locate published scientific studies matching our inclusion criteria. A total of 28 articles not included in Weigelt et al. met criteria for inclusion into this systematic review. Of these 28 studies, 16 were available and met criteria at the time of the previous review, but were mistakenly excluded; and twelve were recently published. Weigelt et al. found quantitative, but not qualitative, differences in face identification in individuals with ASD. In contrast, the current systematic review found both qualitative and quantitative differences in face identification between individuals with and without ASD. There is a large inconsistency in findings across the eye tracking and neurobiological studies reviewed. Recommendations for future research in face recognition in ASD were discussed.

## Introduction

Individuals with autism spectrum disorders (ASD) experience difficulties communicating, which impacts on involvement in social situations [1]. Information from faces is essential during social interactions as it allows inference for recognition and classification [2]. Recognition of faces requires active extraction and encoding of information from the face [2]. It has been suggested that individuals with ASD experience difficulties in facial recognition as a consequence of different strategies in facial processing [3]. Several existing eye tracking studies have discussed the disparity between visual search strategies of faces among individuals with ASD and typically developing (TD) individuals. These results remain disputable partly due to contradictory results between static versus dynamic stimuli [4]. Therefore, a further investigation of the differences of static versus dynamic stimuli in facial recognition is imperative. Another explanation for the difficulties in face recognition experienced by individuals with ASD is the possibility of fundamental neurobiological differences between individuals with and without ASD. Although probable, the precise nature and extent of any neurobiological differences remains undefined [5].

Weigelt et al. [5] recently conducted a review of 91 behavioural studies on face identification abilities of persons with ASD. The review included studies published prior to April 2011that contained experiments on face identity recognition (including face markers) in participants with and without ASD, published in PubMed, The Web of Science, and the database of the National Autistic Society. The authors [5] summarised the studies detailing if and how individuals with ASD processed and identified faces differently *(qualitative differences)*, and what the nature (advantage or disadvantage) and magnitude of any difference might be *(quantitative differences)*. Weigelt et al. [5] presented evidence of quantitative differences in face perception in persons with ASD; namely, two impairments relating to facial memory and eye discrimination. There was no evidence of qualitative differences between how individuals with and without ASD process face information. Weigelt et al. [5]cited both the presence of robust performance in prototypical tasks assessing susceptibility to facial illusions in individuals with ASD, and the paucity of research examining susceptibility to other facial phenomena as proof of this lack of difference. Weigelt et al. [5] included a comprehensive list of 91 studies in their review. The authors did not include 16 studies that were readily available at the time on databases searched for the purposes of including articles into their review. Since April 2011 (the cut-off used in the Weigelt et al. [5] review), an additional 12 studies on this topic have been published. The current systematic review intended to update the evidence base and extend the finding of Weigelt et al. [5], by investigating differences between individuals with and without ASD in behavioural studies of face identification that examined face recognition accuracy and reaction times. Also included in the current review are studies examining visual search strategies and patterns of brain activity during face identification.

## Methods

Seven databases were used to locate published scientific studies matching with the inclusion criteria. These databases were CINAHL Plus, EMBASE, ERIC, Medline, Proquest, PsychInfo and PubMed. The main search terms were “face recognition”, “autism” and “eye tracking”. Using Boolean operators, the following search strategy was used: (“face recogni*” OR “fac* perception” OR “fac* processing” OR “face identi*”) AND (“eye tracking” OR “visual fixation” OR “gaze fixation” OR “eye movement” OR “visual percept*”) AND (autis* OR Asperger OR “autism spectrum disorder”)

Prior to selection of the final published articles, a screening of the titles and abstracts was conducted. After reviewing full text articles, a manual search was conducted through a search of the reference list of the selected articles.

### Inclusion/Exclusion Criteria

Due to ongoing change of the Diagnostic of Statistical Manual of Mental Disorders criteria of ASD throughout the years, the search was limited from year 2000 to May 2013. The latest diagnosis criteria of ASD can be obtained from the Diagnostic of Statistical Manual of Mental Disorders (DSM-5) [1]. Language restrictions were limited to articles published in English. Based on the hierarchy of evidence guidelines in the National Health and Medical Research Council in Australia [6], all types of peer reviewed studies were included in this review. Textbooks were not included.

The current review included the different types of ASD specified in the DSM IV, which included individuals with Autistic disorder, Asperger’s syndrome and Pervasive developmental disorder not otherwise specified (PDD-NOS) [7]. A further inclusion criterion incorporated studies with a comparison group with TD individuals. Studies investigating relatives of individuals with ASD and individuals with other pertinent diagnoses were excluded. Age limits for the participants were not part of the inclusion criteria. Included studies employed static and/or dynamic facial stimuli. Experimental manipulations of face stimuli discussed in Weigelt et al. [5] include the part-whole effect, face inversion effect, face adaptation aftereffects (referred to as face space), Thatcher illusion, simple face perception, fine grained face perception and facial memory were included. Additional exposures included different frequencies of faces, familiar/unfamiliar faces, averted/direct gaze and whole faces with/without non-facial features. The major outcomes of interest were recognition accuracy, reaction times and eye tracking measurements such as number of fixations and fixation durations. Patterns of brain activity as measured by imaging techniques such as functional magnetic resonance imaging (fMRI), electroencephalography (EEG) and magnetoencephalography (MEG) were included as secondary outcomes. Studies included in the previous review by Weigelt et al. [5] that examined emotion recognition and methods involving gender classification were excluded.

An inter-rater reliability score of 0.94 was achieved after evaluation by two reviewers, the first author and an occupational therapy honours student, based on an inclusion and exclusion criteria of a set 50 randomly selected articles. Any discrepancies were resolved with consensus discussion.

### Methodological quality

The methodological quality of each article was assessed using Kmet form (**S1 Table**) [8]. This scoring system is based ona 14 point checklist with sores >80% ranked as strong, 70–80% as good, 50–69% as adequate and <50% as limited. Assessment of possible type II error for studies reporting no statistical differences were conducted using Altman’s Nomogram [9]. Scores and brief descriptions of the methodological quality for included studies are discussed in Appendix B.

### Data Extraction

The extraction of data was based on Cochrane Handbook for Systematic Reviews Section 7.3.a [10]. The headings which were used in the Data Extraction form (**S2 Table**) were citation, level of evidence, ASD population, comparison group, age group, type of stimuli, intervention (referring to experimental manipulation), methods, outcomes and results.

### Data synthesis and analysis

A narrative review approach was applied. The synthesisation and analysis of data were based on themes of recognition accuracy, reaction times, number of fixations, fixation duration and secondary outcomes of brain activation studies fMRI activation, EEG and MEG measures.

## Results

**Fig 1:**
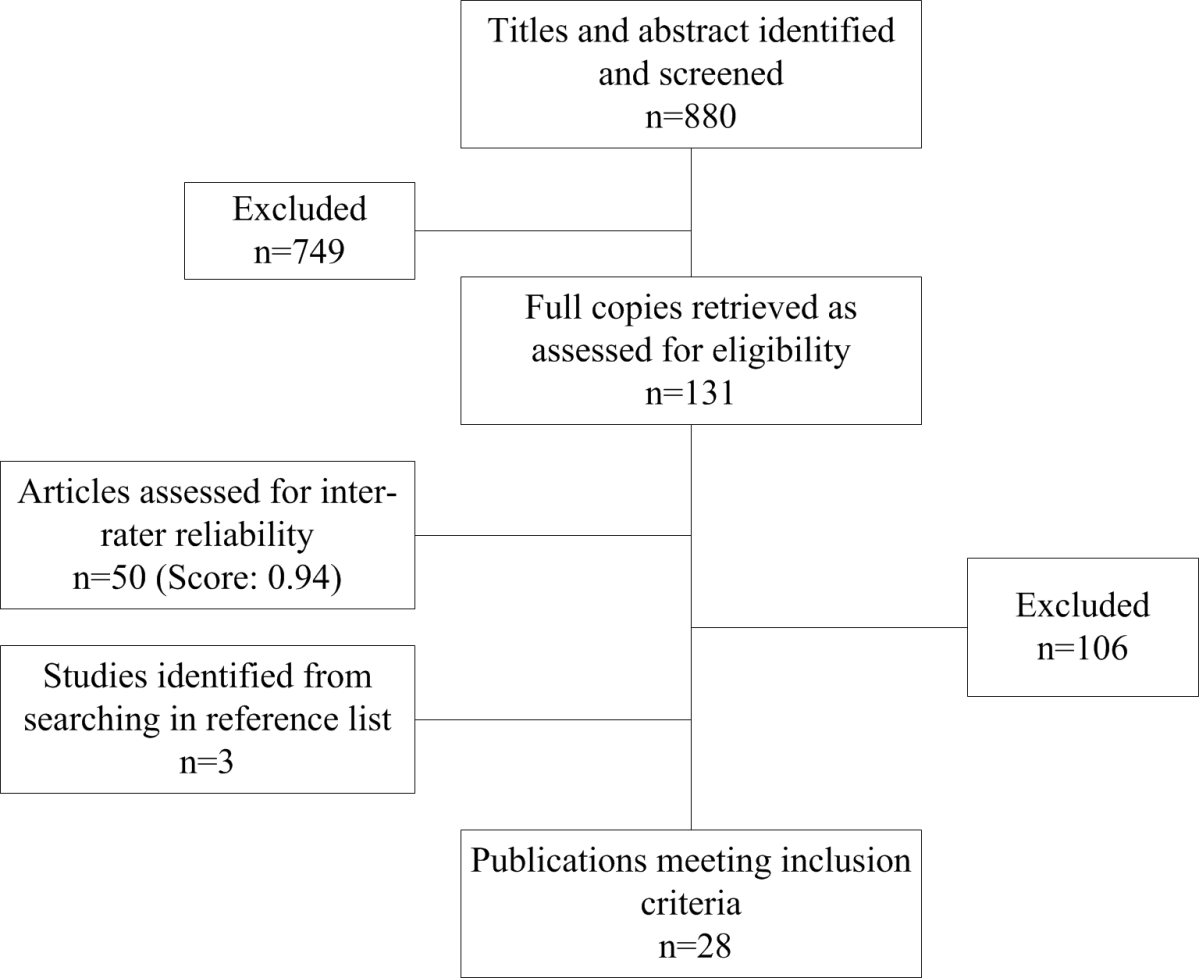
Flow diagram for selection of studies.

Electronic searches located a total of 880 articles. A review of titles and abstracts were conducted and 720 articles were excluded. Evaluation of 131 full text articles was conducted to determine suitability. Based on the exclusion criteria, 106 articles were omitted. Three articles were identified after a manual search of the reference lists resulting in a final inclusion of 28 articles (Fig 1).

The majority of the studies included were case-control studies with an exception of one individual case study. A total of 1329 participants were included. Eight studies utilised eye tracker technology for the measurement of visual search strategies. Ten studies examined differences in patterns of brain activity; EEG was reported in five, fMRI measurements in four, and MEG in one study.

Sixteen employed simple face perception methodology, with stimuli necessitating discrimination between two or more faces. Seven studies used facial memory methods and assed participant’s ability to remember faces after at least a 30 seconds interval between familiarisation and recognition [5]. Several studies used standardised assessments to measure simple face perception or facial memory. These standardised assessments included the Benton Facial Recognition Test, Neuropsychological assessment, and Wechsler memory scale (WMS) 3^rd^ edition-faces subtest. There is currently no evidence on the reliability and validity of the Benton Facial Recognition test, however the Neuropsychological assessment purportedly demonstrates adequate to high reliability and validity [11]. Kent [12] states that although the Wechsler memory scale 3^rd^ edition is a reliable assessment, there may exist issues regarding validity. Four studies utilised the fine grained face perception method, in which face recognition is achieved through successful discrimination between two or more faces with some changes in features of the recognition stimulus [5].

### Quality assessment of studies

The methodological quality of the studies ranged from poor to strong (**S2 Table**). Limitations of the studies included small sample sizes and inadequate descriptions of sampling strategy. The majority of the studies controlled the baseline characteristics for individuals with ASD and the TD individuals. However, the intelligence quotient (IQ) was not controlled in ten studies, which may produce a bias in the obtained results [13–22]. Possible type II error was identified in fourteen studies (**S2 Table**) [13–15, 17, 20–29].

### Intervention (Experimental Manipulation)

Numerous types of stimuli were used in the selected studies with varying levels of experimental manipulations that Weigelt et al [5] refer to as ‘face markers’; from intact faces to reconfigured or obscured faces. Face inversion effects were discussed in six studies where faces were presented upright or inverted [24–26, 30–32]. The inversion effect suggests that face recognition accuracy is reduced when faces are presented upside down [5]. Another seven studies used whole faces, three studies removed the non-facial features (hair and ears), whereas four studies included non-facial features [13, 14, 22, 23, 29, 33, 34]. Unfamiliar and familiar faces were used in five studies [15, 18, 20, 28, 32]. Familiar faces consist of photographs of family members or close friends. The part-whole effect investigates face discrimination by isolating the eyes or the mouths [5]. Two studies described the part-whole effect [17, 27]. Effects of gaze direction on face recognition were measured in three studies [35–37]. Stimuli assessing the influence of gaze direction involve variations in the direction of eye gaze (left, right, direct or closed) towards the participants. Face adaptation after effects (referred to as face space in the Weigelt et al [5] review) were investigated in two studies, where the features of the face were expanded or contracted [30, 31]. Deruelle et al. [16] used faces obscured with different frequency bands filtered to low, high or hybrid. It was proposed that the low filtered frequencies are related to local face processing and high-filtered frequencies involved configural processing [16]. One study examined Thatcher faces in which the mouth or eyes of the faces are inverted [38]. When inverted, Thatcher faces appear ordinary but they are then described as appearing peculiar when upright.

To determine the absence or presence of quantitative or qualitative differences in face recognition in ASD, the studies were categorised under qualitative if the methodology involved face markers in face recognition, such as face inversion effects, part-whole effect, face space and thatcher [5]. Studies involving face discrimination and/or a memory component were classified under quantitative. Overall, a total of eight studies measured face recognition qualitatively [22, 24–26, 30, 31, 38] and 16 studies measured face recognition quantitatively [13, 16, 19, 20, 23, 29, 32–37, 39, 40].

## Outcomes

### Recognition accuracy

A total of 25 studies reported recognition accuracy. Zurcher et al. [38] investigated recognition accuracy in Thatcherized facial stimuli where participants were required to indicate the presence of the Thatcher illusion between two stimuli after cueing to eyes or mouth. Overall performance was better in TD (p<0.05) compared to adults with ASD. When explicitly prompted to view the eyes in upright stimuli, adults with ASD performed significantly better (p<0.05) compared to when they were cued to the mouth.

In a fine grained face perception test (faces in the encoding and familiarisation phases had subtle differences), adults with ASD demonstrated greater difficulty (p = 0.003) in recognizing faces compared with the control group [17]. This study incorporated the part-whole effect on the familiarisation stimuli where faces were divided into different puzzle pieces. The eyes in these puzzle pieces were presented either as a whole or bisected. Individuals without ASD had more correct answers when the eyes were bisected (p<0.001) but similar performance was observed in both groups for whole-eye stimuli. Song et al. [27] employed a part-whole effect stimuli. However, due to insufficient information from the results it was only stated that both ASD and TD group derived information from the eyes in facial recognition tasks. Another fine grained face perception test in Ewing et al. [31] investigated facial discrimination using inversion effects. This study concluded that the inversion effect was present in both groups. However, overall accuracy was significantly better (p<0.05) in both upright and inverted conditions among TD children and adolescents in contrast to the ASD group. The reduced recognition accuracy in this study was also surprisingly extended when viewing cars. This indicates that children and adolescents with ASD demonstrated recognition difficulties in faces, as well as in objects. Ewing et al. [30] investigated face adaptation aftereffects on recognition among children with ASD and matched TD peers. Aftereffects to upright faces were significantly reduced (p<0.05) in children with ASD relative to controls. This was not observed across inverted faces and cars.

Seven studies investigated facial memory recall accuracy and employed measures with or without a standardised assessment. Out of the 28 included studies, three studies used Wechsler memory scale scores for both immediate and delayed memory [32, 39, 40]. All three studies report significantly poorer accuracy in individuals with ASD. The Neuropsychological is another standardised assessment of facial memory. This measure was used in two studies and the results of these studies were conflicting. In one study, children in the TD group were significantly more accurate in both immediate and delayed recognition [19]. However, Tehrani-Doost et al. [21] recorded no significant differences in facial memory accuracy. The conflicting results could be attributed to a lack of controlling for IQ in the Tehrani-Doost et al. [21] study. Three studies investigated facial memory without standardised assessments [28, 31, 37]. Recognition tasks in these three studies involved participants being exposed to a series of facial stimuli, each image for a period of three to eight seconds. The participants were then shown another set of images and they were required to discriminate which of these stimuli had been presented in the previous encoding condition. Statistical significance was achieved in Ewing et al. [31] (p<0.05) and Wilson et al. [37] (p<0.001), with children with ASD poorer at accurately recognizing of previous stimuli. Incongruously, Sterling et al. [28] failed to find a significant difference between individuals with and without ASD.

Benton Facial Recognition Test is a standardised assessment which measures simple face perception and this was used in two studies [25, 32]. The ASD group demonstrated poorer recognition accuracy (p<0.01) compared to TD peers in both studies. Snow et al. [33] and Trepagnier et al. [22] employed a similar procedure which presented participants with a series of images in the familiarisation phase, followed by a slight delay. Participants were then required to indicate whether a face or object had been previously presented in the familiarisation phase [22, 33]. Both of these studies found significant differences (p<0.05) in recognition accuracy, with poorer facial recognition observed for individuals with ASD compared to object recognition. The TD group in Snow et al. [33] recorded similar accuracy to both faces and objects whereas in Trepagnier et al. [22] the ASD group exhibited largely superior performance in object recognition compared to TD controls. In a different study, Kylliainen et al. [35] recorded better accuracy in the TD group. However, object recognition accuracy was similar for both TD and ASD participants. This study employed a simultaneous recognition strategy, which required the participants to respond when the stimulus was a repetition or different [35]. Wilkinson et al. [29] and Bradshaw et al. [13] used a similar procedure with the addition of a slight delay between exposure and recognition stimuli. Bradshaw et al. [13] reported poorer recognition accuracy among the group with ASD. In Wilkinson et. al. [29], recognition accuracy was based on the comparison of recall awareness measured with a three-point rating scale of *‘certain’*, *‘somewhat certain’* and *‘guessing’*. It was expected that when an individual is *‘certain’* the face recognition accuracy improves. This is present among TD children as performed better when they were ‘certain’ (p<0.01). However, memory awareness accuracy was improved when children with ASD indicated ‘guessing’ (p<0.05) in comparison to TD children. This indicates a possible delay in their memory awareness. Recognition accuracy was similar between both groups when they were *‘somewhat certain’*. When accuracy was compared with adults with ASD, recall awareness was better in comparison to children with ASD. Due to the younger population used in Chawarska and Volkmar [23], recognition accuracy was measured using a comparison of fixation duration of familiar and novel stimuli. The younger ASD group demonstrated poorer facial recognition accuracy despite the older ASD group performing at a similar level to TD controls.

Deruelle et al. [16] studied the effect of different frequencies of faces using a two alternative forced choice paradigm. Overall results revealed that both groups had higher number of correct responses in low-pass frequencies faces (p<0.01) in comparison to the high-pass frequencies. Typical individuals had more errors in high pass conditions, while the ASD group were efficient in facial recognition in high and low facial frequencies.

As shown in Table 1, only seven studies did not demonstrate statistically significant differences in recognition accuracy [14, 20, 21, 26, 28, 29]. However, differences in recognition accuracy between children with ASD and TD individuals were difficult to interpret in Wilson et al. [34] due to high variance in the ASD group. Therefore, some children with ASD displayed similar facial recognition accuracy while others had significantly lower scores compared to controls [34].

**Table 1:** Face recognition accuracy results distribution for ASD in comparison to TD.

Studies	No differences	Reduced	Better
Bradshaw et al. [13]		x	
Chawarska & Shic [14]	x		
Chawarska & Volkmar [23]		x	
Deruelle et al. [16]	x (in low-pass frequencies)		x (in high-pass frequencies)
Ewing et al. [31]		x	
Ewing et al. [30]		x	
Falkmer et al. [17]		x	
Kleinhans et al. [24]	x		
Kylläinen et al. [35]		x	
McPartland et al. [39]		x	
McPartland et al. [25]		x	
Parish-Morris et al. [36]		x	
Pierce & Redcay [20]	x		
Reed et al. [26]	x		
Kuusikko et al. [19]		x	
Snow et al. [33]		x	
Sterling et al. [28]	x		
Tehrani-Doost et al. [21]	x		
Trepagnier et al. [22]		x	
Webb et al. [40]		x	
Webb et al. [32]		x	
Wilkinson et al. [29]	x (adults)	x (children)	
Wilson et al. [34]	x	x	
Wilson et al. [37]		x	
Zurcher et al. [38]		x	

Majority of the studies achieved statistical differences, which signify that TD individuals performed better in facial recognition tasks in comparison to individuals with ASD. Overall, the quantitative measurements in face recognition were reported in 16 studies. Three of these studies reported mixed results. With the exclusion of the mixed results studies, a total of 11 studies out of the 13 quantitative studies (85%) reported reduced face recognition accuracy among individuals with ASD. Qualitatively, a similar pattern was observed as six out of the eight qualitative studies (75%) reported poorer face recognition in individuals with ASD. Therefore, the studies reviewed reported both qualitative and quantitative differences were observed in face recognition individuals with ASD.

### Reaction time

Six studies included reports of reaction time. In a Benton Facial Recognition test, Tehrani-Doost et al. [21] recorded similarity in reaction times in TD individuals and ASD individuals. The same result was obtained when unfamiliar and familiar faces were presented [20, 28]. However, children with ASD demonstrated slower reaction times when unfamiliar faces were presented [20]. A contrasting result was obtained by Kleinhans et al. [24] who concluded that reaction times were significantly slower among adults with ASD during the first trial (p<0.03). This result was non-significant during the second trial [24]. In Thatcherized stimuli, adults with ASD reacted faster than TD individuals to inverted stimuli [38]. In an individual case study comparing reaction times in human faces, cartoons and objects, overall reaction times were slower for a child with ASD [18]. Conversely, the child with ASD was able to react faster when his favourite cartoon was presented in comparison to faces and objects. A TD control child had quicker reaction times for human faces and his favourite cartoon but slower reactions were recorded for objects and the favourite cartoon.

As shown in Table 2, most studies suggest that there are no differences in reaction time in face recognition tasks.

**Table 2:** Reaction time results distribution for ASD in comparison to TD.

Studies	Grelotti et al. [18]	Kleinhans et al. [24]	Pierce & Redcay [20]	Sterling et al. [28]	Tehrani-Doost et al. [21]	Zurcher et al. [38]
**Similar**			x	x	x	
**Slower**	x	x	x (only in unfamiliar faces)			x (only inverted conditions)

### Fixation duration

Measurements of fixation duration were discussed in all seven eye tracking studies. Bradshaw et al. [13] found no significant differences between children with ASD and TD children in fixation duration. Interestingly, the same result was concluded in a study by Parish-Morris et al. [36] using dynamic facial stimuli. Although face recognition was significantly correlated with facial attention (p = 0.02), no significant differences in fixation duration were found between both groups.

Chawarska & Shic [14] compared fixation duration in two different age groups (two and four years old). Fixations on inner features of the face were significantly reduced in the older ASD group in comparison to both controls (p<0.014 and p<0.015) and younger ASD group (p<0.034). However, both ASD groups demonstrated increased fixation on outer faces compared to controls (p<0.027). Overall, fixation durations on the eyes decreased significantly with age (p<0.045) indicating that preference in looking at the eyes reduced with age. Fixation duration on the mouth was significantly reduced in children with ASD (p<0.001) compared to TD children.

Overall, fixation duration comparisons in Sterling et al. [28] revealed increased duration among controls in the eyes across both familiar and unfamiliar conditions. Conversely, in both conditions, fixation duration on the mouth was similar for both ASD group and controls [28]. When unfamiliar facial stimuli were presented, fixation durations on eyes and mouth were increased in both groups [28]. This result contradicts that of Snow et al. [33] as TD individuals spent longer time fixating on core features of the face (eyes, mouth and nose) compared to individuals with ASD (p<0.01).

Outcomes of fixation duration in Falkmer et al. [17] compared the differences between puzzle-pieced and whole-face recognition stimuli. Since there were a variety of presentations of the eyes (bisected and whole) in the puzzle-pieced stimuli, it was concluded that TD adults spent more time on the eyes in both intact and halves conditions. Increased fixation duration on the eyes and the mouth puzzle pieces was demonstrated in TD adults. However, adults with ASD spent significantly longer time on eyes and other features besides the nose and mouth in whole-face recognition stimuli.

A study by Wilson et al. [37] found no significant differences between groups when three main features of the face, eyes, mouths and noses were individually compared. However, comparison as a whole achieved statistical significance (p = 0.04), ASD group spent less time on these core features.

Due to the differences in the classification of the areas of interests across the seven identified studies in fixation duration, studies which classified the individual features of the face, more specifically the ‘eyes’ and ‘mouth’, were retrieved for further comparisons. Five studies were reported the results of fixation durations in the ‘eyes’ and ‘mouth’. Majority of these studies (eyes = 3, mouth = 4) reported similar fixation durations towards the ‘eyes’ and ‘mouth’, as illustrated in Table 3.

**Table 3:** Fixation duration results distribution for ASD in comparison to TD.

Studies		Bradshaw et al. [13]	Chawarska & Shic [14]	Falkmer et al. [17]	Parish-Morris et al. [36]	Snow et al. [33]	Sterling et al. [28]	Wilson et al. [39]
**Eyes**	Similar		x			x		x
	Longer			x (only in the recognition phase)				
	Shorter			x (only in the encoding phase)			x	
**Nose**	Similar		x			x		x
	Longer							
	Shorter							
**Mouth**	Similar			x (only in the recognition phase)		x	x	x
	Longer							
	Shorter		x	x (only in the encoding phase)				
**Other**	Similar			x (only in the encoding phase)				
	Longer			x (only in the encoding phase)				
	Shorter							
**Inner features**	Similar		x (only in younger ASD group)					
	Longer							
	Shorter		x (only in older ASD group)					x
**Outer features**	Similar							x
	Longer		x					
	Shorter					x		
**Stimuli type (faces vs objects)**	Less in faces				similar			

### Number of fixations

Four eye tracking studies measured number of fixations. In a part-whole effect study, Falkmer et al. [17] divided the familiarisation facial stimuli into different puzzle pieces. Fixations on the eyes in the puzzle pieces were significantly more prevalent in control adults (p = 0.004) and fixations on other areas of the face were low (p = 0.002) in comparison to adults with ASD. These results were similar in the recognition stimuli where controls exhibit more fixations on the eyes (p<0.001) and mouth (p = 0.049). However, adults with ASD displayed a preference for parts other than the eyes and mouth (p<0.001).

Examination of number of fixations in primary regions of the face (eyes, nose and mouth) revealed that both ASD and TD groups exhibit higher preference for fixating on the eyes [33]. There were no significant differences between the two groups. Number of fixations outside of the primary regions was significantly greater among TD adults (p<0.05) compared to ASD participants.

Interestingly, Sterling et al. [28] concluded that adults with ASD did not differ in number of fixations when viewing both familiar and unfamiliar stimuli. However, the TD group exhibited a higher number of fixations (p<0.02) in the unfamiliar faces condition compared to the familiar faces [28]. Differences between the two groups did not achieve significance. The control group exhibited a significantly (p<0.02) higher number of fixations in the eye and mouth regions in comparison to the adults with ASD across all the stimuli presented [28].

Trepagnier et al. [22] demonstrated that individuals with ASD had reduced percentage in fixations of the central face region for both familiarisation and recognition stimulus. However, this result is susceptible to bias due to small sample size and reduced reliability of eye tracking measurements.

In summary, individuals with ASD demonstrated a decreased in the number of fixations towards the ‘eyes’ or the inner features of the face, i.e., eyes nose and mouth among the group with ASD. However, there is a large inconsistency in findings among the analysis of number of fixations towards the ‘mouth’. A summary of the results are presented in Table 4.

**Table 4:** Number of fixations results distribution for ASD in comparison to TD.

Studies		Falkmer et al. [17]	Snow et al. [33]	Sterling et al. [28]	Trepagnier et al. [22]
**Eyes**	Similar		x		
	Increased				
	Decreased	x (encoding and recognition phase)		x	
**Nose**	Similar		x		
	Increased				
	Decreased				
**Mouth**	Similar	x (only in the encoding phase)	x		
	Increased				
	Decreased	x (only in the recognition phase)		x	
**Other**	Similar				
	Increased	x (only in the encoding phase)			
	Decreased	x (only in the recognition phase)	x		
**Inner features**	Similar				
	Increased				
	Decreased				x
**Stimuli type (faces vs objects)**			Decreased in faces		

### fMRI activation

The areas of interest investigated in Pierce and Redcay [20] were the amygdala, fusiform face area, posterior and anterior cingulate. Group differences in activation between TD individuals and children with ASD were not observed in the amygdala and anterior cingulate. However, a significant difference in activation of the fusiform was observed bilaterally in controls, with individuals with ASD exhibiting only right hemispheric activation of the fusiform. The posterior cingulate activation was significantly decreased in children with ASD when familiar stimuli were presented in comparison to TD children. Activation of the fusiform and amygdala were present in both groups. However, controls exhibited additional activation of anterior and posterior cingulate areas during the familiar faces condition. A different outcome was obtained by Kleinhans et al. [24] who investigated recognition of faces with neutral unfamiliar faces in upright and inverted conditions in adults with and without ASD. Statistically significant differences in activation were not observed in the fusiform gyrus when groups were compared. However, adults with ASD exhibited significantly reduced activity in the amygdala bilaterally (p<0.05).

Grelotti et al. [18] concluded that activation of the fusiform gyrus and amygdala were present when viewing cartoons but this was decreased during in an unfamiliar faces condition in a child with ASD. Conversely, greater activation in the fusiform gyrus and amygdala were observed in a TD adolescent when viewing unfamiliar faces in comparison to viewing objects.

fMRI activation was analysed differently in Zurcher et al. [38] as it was based on the response to prompt towards eyes or mouth in Thatcherized stimuli. Both groups showed similar activation to the fusiform and lateral occipital cortex (two of the cortical areas analysed) when prompted to the eyes and mouth. Adults with ASD showed heightened activation when prompt was directed towards the eyes in both one of the cortical area (pars opercularis of inferior frontal gyrus) in the inverted condition and subcortical areas (amygdala and pulvinar nucleus in thalamus) in the upright conditions which were shown to be involved in face processing.

In conclusion, specific differences in fMRI activation were inconclusive, as presented in Table 5.

**Table 5:** fMRI results distribution for ASD in comparison to TD.

Studies		Grelotti et al. [18]	Kleinhans et al. [24]	Pierce & Redcay [20]	Zurcher et al. [38] (cued to the eyes)
**Fusiform gyrus**	Heightened				x
	Reduced	x		x (in unfamiliar faces)	
	Similar				
**Amygdala**	Heightened				x
	Reduced	x	x		
	Similar			x	
**Others**	Heightened				x (lateral occipital cortex, pars opercularis of inferior frontal gyrus and pulvinar nucleus in thalamus)
	Reduced			x (posterior cingulate- in familiar faces)	
	Similar			x (anterior cingulate)	

### EEG/ERP measures

Previous research has demonstrated that the N170 component is involved in face processing [41]. Bentin et al. [42] first described the behaviour of the N170 on TD individuals which was evoked when human faces were presented and absence during animate and inanimate nonface stimulations (e.g. faces of animals, human hands, cars and house furniture), The study revealed that distorted human faces evoked N170 which has the same amplitude as normal faces. When the facial landmarks were presented in isolation, they found out that the eye regions elicited an N170 that was much larger than the entire face. They also reported that the nose and lip regions elicited a slightly delayed N170 (about 50 msec later). The study also revealed that the N170 was delayed when faces are inverted with no changes in the amplitude. Three studies included a discussion of N170 among individuals with autism and their typically developing counterparts [25, 32, 39].

In McPartland et al. [39], individuals with ASD produced longer N170 latency in response to faces compared to TD individuals (p<0.01). However, a significant difference between both groups in N170 amplitude was not discovered. McPartland et al. [25] compared N170 latencies between faces and houses and concluded that TD adults had smaller N170 latencies in face conditions compared to adults with ASD. When the effects of inversion were examined, N170 amplitude of the ASD group was reduced for inverted faces relative to the TD group. Between faces and houses, N170 amplitude was displayed in both hemispheres but this was more evident in the right hemisphere among TD individuals. Webb et al. [32] did not report significant differences in N170 latency between groups when upright and inverted faces where shown to adult subjects. Differences in N170 amplitude to upright faces did not reach significance but more negative amplitude was observed in TD individuals for inverted faces compared to adults with ASD.

It has been previously suggested that the ERP, P1 is associated with early visual processing modulated by attention [43]. Both McPartland et al. [25] and Webb et al. [32] recorded no statistical significance between groups where both groups exhibited more negative P1 amplitude in faces in comparison to houses. The group of TD individuals had increased P1 amplitude in inverted faces in comparison to upright conditions [32]. This was not present in the ASD group. However, McPartland et al. [25] recorded no differences in both groups as greater amplitude in both inverted and upright faces were observed. No significant differences in P1 latencies were observed in either study, although McPartland et al. [25] reported that both group demonstrated reduced latencies for faces [25, 32].

Additional discussion of other ERPs like P2, N250 and face-N400 components can be found in Webb et al. [40] and all are purportedly involved in identity processing. Webb et al. [40] found no differences in P2, N250, N400, P1 and N170 amplitudes or latencies between adults with and without ASD. P400 and Nc amplitudes are purportedly associated with face processing and recognition involving memory [15]. Dawson et al. [15] measured differences in P400 and Negative Component (Nc) amplitudes between TD children and children with ASD. Analysis of familiar versus unfamiliar faces revealed that TD children exhibited larger P400 and Nc amplitude in unfamiliar faces. Significant differences in P400 and Nc amplitude between familiar and unfamiliar faces were not observed in children with ASD.

As demonstrated in Table 6, results of EEG measures were varied perhaps due to the varying experimental protocols used in these experiment and the varying physiological conditions of the ASD subjects. Thus, a definite conclusion of differences in EEG or ERP components could not be made.

**Table 6:** EEG results distribution for ASD in comparison to TD.

Studies		Dawson et al. [15]	McPartland et al. [39]	McPartland et al. [25]	Webb et al. [40]	Webb et al. [32]
**N170 amplitude**	Increased					x (inverted faces)
	Decreased					
	Similar		x		x	x
**N170 latency**	Increased		x	x (faces versus houses)		
	Decreased			x (inverted faces)		
	Similar				x	x
**P1 amplitude**	Increased					
	Decreased					
	Similar	x			x	
**P1 latency**	Increased					
	Decreased					
	Similar			x	x	x
**Others**	Increased	x (P400 and Nc amplitude)				
	Decreased					
	Similar				x (P2, N250, N400)	

### MEG

Kylläinen et al. [35] studied face and gaze processing in 7–12 year old children with and without ASD using MEG. The study found that face and gaze processing in the ASD group followed a trajectory somewhat similar to that seen in TD children; but with subtle differences. For example, when participants were presented with face stimuli at 100 ms, a strong activity over posterior brain regions was noted in both groups. A response at 140 ms to faces observed over extrastriate cortices (analogous to the N170 in adults) was weak and bilateral in both groups and somewhat weaker (approaching significance) in the ASD subgroup.

Overall, while the studies using imaging techniques demonstrated some patterns in brain activity (both similarities and differences between ASD and TD as shown in Tables 5 and 6), the exact relationships in qualitative or quantitative differences remains unknown.

## Discussion

Weigelt et al. [5] concluded that face identity perception appears to be *qualitatively* similar between individuals with and without ASD, given that many of the hallmarks of face processing (or face markers) were apparently intact in individuals with ASD. However, two specific *quantitative* differences in face identity perception are described; namely a face-specific reduction in accurate recall and eye-specific discrimination deficit for individuals with ASD. The conclusions of the synthesis of studies not discussed by Weigelt et al. [5] and published since April 2011 are simultaneously congruous and incongruous with those presented by Weigelt et al. [5]. The findings of the current systematic review indicate that individuals with ASD indeed exhibit poorer facial recognition accuracy relative to their TD peers, thus supporting the conclusions regarding quantitative differences in Weigelt et al. [5]. However, in contradiction to Weigelt et al. [5], this review of missing and recently published studies has found reduced face identity perception for many face markers in individuals with ASD. This indicates that more recent research suggests these hallmarks of face processing are in fact not intact; and qualitative differences between individuals with and without ASD may exist. A further evaluation of reaction times indicates that individuals with ASD and TD individuals responded at similar speeds during face recognition tasks. ASD specific differences in brain activation could not be inferred due to the variety of results presented.

This systematic review inherited the theoretical framework adopted by Weigelt et al. [1], who provided the distinction of *quantitative* and *qualitative* face recognition processes in ASD. Studies involving different “face markers”, i.e., face inversion effects, face space and Thatcher illusion were classified under *qualitative* studies [1]. *Quantitative* studies included studies with (1) a working memory component, (2) simple visual perceptual discrimination between different faces, (3) perceptual discrimination of similar faces with subtle differences in face features, and (4) standardized face recognition assessments [1]. However, it is acknowledged that the distinction between *qualitative* and *quantitative* differences can be ambiguous, which could suggest a possible inherent methodological limitation of this systematic review to address face recognition processes. Ideally, an alternative framework should have been developed for this purpose but that would then have made the extension of the previous review impossible.

The comprehensive review by Weigelt et al. [5] chose to address only behavioural studies on this topic. The current review included studies that used eye tracking, fMRI, EEG and MEG in order to review possible neurobiological correlates with a potentially causal role in the observed behavioural differences between indiviuals with and without ASD. Despite there being numerous likely candidates for neurobiological differences, the current review found little consistency across studies or imaging techniques. At first glance, the derivative of the exact differences in visual search strategies can be challenging due to the inconsistencies reported in literature. However, eye tracking comparisons specifically on the individual face features revealed similar fixation durations but a decreased number of fixations towards the ‘eyes’ among individuals with ASD compared to TD individuals. Additionally, both eye tracking measurements revealed no differences relative to the ‘mouth’ in both of the population groups. The ‘eye’ fixation durations interpretation in this systematic review is contradictory to a recent review in general face processing in ASD conducted by Papagiannopoulou et al. [44]. The heterogeneity of results in eye tracking studies could be partly due to the variety in the analytical procedures (univariate versus multivariate), classification of areas of interest, eye tracking technology and face recognition measurements, i.e. *qualitative* or *quantitative* stimuli.

However, caution should be given to the interpretation of the eye tracking and neurobiological results in this systematic review due to the small number of articles included (eye tracking = 8, neuroimaging = 4 and neurophysiological = 5). Due to this, definitive *qualitative* and *qualitative* face recognition conclusions in visual search strategies and brain activation processes cannot be derived. However, the conclusion from this systematic review was based on the comparisons of 734 participants (55.2% of the 1329 participants as demonstrated in Table 7) from the eye tracking, neuroimaging and neurophysiological face recognition articles in ASD. Thus, further behavioural and neurobiological studies are required before definitive conclusions can be made regarding ASD-specific deficits or differences. Additionally, while this systematic review included neurobiological studies in face recognition these were only discussed as secondary outcomes. As this systematic review builds upon Weigelt et al. [5] review on the behavioural outcomes in face recognition tasks in ASD, the focus on this systematic review is primarily on the behavioural and eye tracking outcomes in face recognition among individuals with ASD. As a result of this, the search terms used prioritised the primary outcomes (behavioural and eye tracking measurements) of this review and consequently underrepresent the imaging and EEG studies on face perception in ASD. For further face processing reviews or summary of neuroimaging and neurophysiological processes in ASD; refer to Harms et al. [45], Luckhardt et al. [46]) and Perlman et al. [47].

**Table 7:** Percentages of participants in eye tracking and neurobiological studies in this systematic review.

Face recognition outcomes	Number of studies	Number of participants/Percentages
**Eye Tracking**	8	385 (29.0%)
**fMRI**	4	98 (7.3%)
**EEG**	5	251 (18.9%)
**Total**	17	734 (55.2%)

The main limitation in the majority of the studies reported was low sample size. However, this is a common occurrence across most research due to difficulties relating to recruitment. Sampling strategy could be determined in a minority of studies and ten studies revealed possible confounding factors due to insufficient control of IQ at baseline. Possible type II errors were identified in fourteen studies (**S2 Table)**. Despite limitations, it can be stated that most studies were scored as having good to strong quality based on Kmet forms. Furthermore, casecontrol study design is indeed the most appropriate design to investigate differences between individuals with ASD and their TD controls.

Two specific face markers discussed by Weigelt et al. [5], left side bias and composite faces, were not used in the studies included in the current review. Similarly only one study used the Thatcher illusion and two used face space in face identification. The paucity of research examining these face markers was also observed by who discussed the necessity for future research in these specific phenomena. Although studies involving individuals with PDD-NOS were included in the current review, the term “PDD-NOS” was not included in the search strategy. Thus, it is possible that the PDD-NOS population could be underrepresented in our review. The selection of articles was limited to electronic published peer reviewed journal articles. Manual search of grey literature was not conducted. However, this review offers a comprehensive search strategy from seven different databases.

Differences in face recognition in static versus dynamic stimuli could not be derived from this review as only one study used dynamic stimuli to measure differences in facial recognition in individuals with ASD and their TD peers. The prevalent face processing evidence reveals that there is a difference in visual search strategies in dynamic and static methodologies [4, 48]. Studies suggest that in dynamic stimuli participants with ASD fixated less on the eyes and more on the mouths or other parts of the body [4, 48]. However, this was not reflected in a study by Falkmer, Bjaellmark, Larsson, and Falkmer [49] who found no differences in visual search strategies between real dynamic and static stimuli. Thus, future research could prioritise the use of dynamic stimuli to further investigate the possible differences in facial recognition in static versus dynamic stimuli.

It is possible that the lack of definitive conclusions regarding visual search strategies and brain activation could be due to differences between children, adolescents and adults with ASD. Only three studies included in this review directly compared facial recognition abilities between children and adults with ASD [14, 23, 29]. All three studies suggest possible differences between children and adults with ASD. Thus, the question for future research is; do visual strategies in individuals with ASD change differently in comparison with TD individuals over time and how does this impact on face identification abilities? If it does, when in the developmental trajectory does changes occur?

The results from the current review contradict the findings of the review undertaken by Weigelt et al. [5] to suggest that individuals with ASD exhibit both qualitative and quantitative differences in face identity processing. However, the question whether these difficulties are due to ASD specific differences in visual strategies or brain activation during face identification tasks remains unanswered. The contradictory findings and the indefinite answers in face recognition in ASD concluded in this systematic review reflect the complexity of autism as a condition. It is recommended that future research should address these inconsistencies through careful consideration of the heterogeneous measurements in face recognition, e.g., quantitative and qualitative measurements, and a control for sampling characteristics such as diagnosis, age and IQ matching.

## Supporting Information

S1 Table Kmet form. (DOCX)

S2 Table Data Extraction Form. (DOCX)

S3 Table Prisma Checklist. (PDF)
