# Supplementary material for: Face Recognition and Visual Search Strategies in Autism Spectrum Disorders: Amending and Extending a Recent Review by Weigelt et al
Source: PLoS One. 2015 Aug 7;10(8):e0134439. doi: 10.1371/journal.pone.0134439 (PMC4529109; doi:10.1371/journal.pone.0134439)
Supplement: S1 Table — (DOCX) [file pone.0134439.s001.docx]

|  | Criteria | YES (2) | PARTIAL (1) | NO (0) | N/A |
| --- | --- | --- | --- | --- | --- |
| 1 | Question/objective sufficiently described |  |  |  |  |
| 2 | Study design evident and appropriate? |  |  |  |  |
| 3 | Method of subject/comparison group selection or source of information/input variables described and appropriate? |  |  |  |  |
| 4 | Subject (and comparison group, if applicable) characteristics sufficiently described? |  |  |  |  |
| 5 | If interventional and random allocation was possible, was it described? |  |  |  |  |
| 6 | If interventional and blinding of investigators was possible, was it reported? |  |  |  |  |
| 7 | If interventional and blinding of subjects was possible, was it reported? |  |  |  |  |
| 8 | Outcome and (if applicable) exposure measure(s) well defined and robust to measurement/ misclassification bias? |  |  |  |  |
| 9 | Sample size appropriate? |  |  |  |  |
| 10 | Analytic methods described/justified and appropriate |  |  |  |  |
| 11 | Some estimate of variance is reported for the main results |  |  |  |  |
| 12 | Controlled for confounding? |  |  |  |  |
| 13 | Results reported in sufficient detail? |  |  |  |  |
| 14 | Conclusions supported by the results? |  |  |  |  |

**S1 Table.** Kmet form. Retrieved from, L. M., Lee, R. C., & Cook, L. S. (2004). Standard quality assessment criteria for evaluating primary research papers from a variety of fields. Alberta, Canada: Alberta Heritage Foundation for Medical Research
