# Supplementary material for: Face Recognition and Visual Search Strategies in Autism Spectrum Disorders: Amending and Extending a Recent Review by Weigelt et al
Source: PLoS One. 2015 Aug 7;10(8):e0134439. doi: 10.1371/journal.pone.0134439 (PMC4529109; doi:10.1371/journal.pone.0134439)
Supplement: S2 Table — (DOCX) [file pone.0134439.s002.docx]

| Citation, level of evidence | ASD population | | | Control group | | Age group | Type of stimuli | Intervention | Method | Outcomes/ Result | Methodological quality |
| --- | --- | --- | --- | --- | --- | --- | --- | --- | --- | --- | --- |
|  | ***Diagnosis*** | ***N*** | ***Mean age (SD)*** | ***N*** | ***Mean age (SD)*** |  |  |  |  |  |  |
| Bradshaw et al. [13], level III | ASD | 21 | 39.0 (10) M | 21 | 36 (7) M | CH | Static | Whole faces | **Eye tracking. Simple face perception-** slight delay VPC | **Recognition accuracy-** no significant differences **Fixation duration**- no significant differences | Good quality (score= 17/24). Reliable and valid instrument used. Sampling strategy identified. An inclusion/exclusion criterion of the ASD group was not sufficiently described. Investigators were not blinded. Low sample size. Possible Type II error calculated (d=0) |
| Chawarska & Shic [14], level III | Autism, PDD-NOS | 44 | Age1: 26.9 (6.2) M, Age2: 46.4 (6.4) M | 30 | Age 1: 26.3 (6.5)M. Age 2: 46.3 (4.3) M | CH | Static | Whole faces | **Eye tracking Simple face perception-** slight delay VPC | **Recognition accuracy**- no significant differences **Fixation duration**- ↓ in older ASD in inner facial features across all groups. ASD ↑time on the outer face (p<0.027). Older ASD ↑ time on non-face features than young ASD group (p<0.001) and TD (p<0.018 & p<0.052). ↓ in the mouth among children with ASD (p<0.001). | Strong quality (score: 21/24). Reliable and valid instrument used. Sampling strategy identified. Blinding of investigators not described. Reported significant differences in verbal level of functioning (possible confounding factor). Possible Type II error calculated (d=0.02) |
| Chawarska & Volkmar [23], level III | ASD | A:14 B: 15 | A: 27.9 (4.9) M B: 45.9 (6.7) M | TD- A: 10 B: 13DD- A: 16B: 10 | TD- A: 19.7 (4.7) M, B: 38.0 (8.2) M, DD- A: 23.7 (6.7) M, B: 39.5 (6.7) M | CH | Static | Whole faces and monkey faces | **Simple face perception-** Slight delay VPC | **Recognition accuracy**- Younger ASD (2 years) group showed no evidence in face and monkey recognition. ↑in faces was demonstrated in TD. Older ASD group (3-4 years) ↑in recognition compared to younger ASD and performed similarly to TD. | Strong quality (score: 20/22). Reliable and valid instrument used. Sampling strategy is sufficiently described. Provided good inclusion/exclusion criteria. Controlled for baseline characteristics. Small sample size. Possible Type II error calculated (d=0.54) |
| Dawson et al. [15], level III | Autism, PDD-NOS | 34 | 28.1 (9.6)M | TD: 19, DD: 16 | TD: 45.4 (6.2) M, DD: 44.8 (4.9) M | CH | Static | Familiar/ Unfamiliar faces and objects | **EEG** | **EEG measures-*P400:*** TD showed ↑ P400 amplitude to unfamiliar than familiar face (p<0.05) but ASD showed no significant differences. **Nc:** TD ↑amplitude to unfamiliar vs familiar faces (p<0.05) but no differences in ASD group. | Strong quality (score: 19/22). Reliable and valid instrument used. Sampling strategy is sufficiently described. Provided good inclusion/exclusion criteria. Sample size was appropriate for ASD group but inadequate in the TD group. Reported significant differences in mental age (confounding factor). Possible Type II error calculated (d=0.46). |
| Deruelle et al. [16], level III | Autism, AS | 17 | 10 Y 5 M (2 Y 8 M) | 17 | 10 Y 5 M (2 Y 8 M) | CH | Static | Different frequencies of faces (high, low and hybrid) | **Simple face perception-** simultaneous VPC | **Recognition accuracy**- Overall, ↓ in high frequencies faces compared to low (p<0.01). But TD group ↑ in low frequencies condition (p<0.05). | Strong quality (score: 18/22). Reliable and valid instrument used. Provided good inclusion/exclusion criteria. Sampling strategy not described. Small sample size. Baseline characteristics of participants were not controlled (IQ). |
| Ewing et al. [31], level III | Autism, AS, PDD-NOS | 40 | 138.2 (26.5) M | 40 | 139.5 (32.9) M | CH & ADO | Static | Inversion effects- faces and cars, Face space | **Facial memory-** remember 10 images (30s), identify old-new, **Fine grained perception** (face space) - identify which image looked similar to the test stimulus. | **Recognition accuracy-** face discrimination (p<0.05) and memory (p<0.01) was ↑in TD. | Strong quality (score: 22/22). Instrument tested for reliability and validity. Sampling strategy and inclusion/exclusion criteria clearly identified. Controlled for confounding factors. Good sample size. |
| Ewing et al. [30], level III | Autism, AS, PDD-NOS | 29 | 141.9 (26.7) M | 29 | 141.2 (32.8) M | CH & ADO | Static | Inversion effects- faces and cars. Face space | **Fine grained perception (face space)-** label with “squished in” or “squished out”, **Simple face perception**- respond when same stimulus appear consecutively | **Recognition accuracy-** affereffects ↓ in children with ASD in upright faces. | Strong quality (score: 21/22). Reliable and valid instrument used. Sampling strategy is sufficiently described. Inclusion/exclusion criteria of participants were not sufficiently described. |
| Falkmer et al. [17] , level III | AS | 24 | 29.0 (10.8) Y | 24 | 28.9 (10.7) Y | ADO | Static | Part whole effect- face cut up into six puzzle pieces (eyes intact or cut into halves) | **Eye tracking Fine grained perception-** featural changes were made to the recognition stimulus | **Recognition accuracy-** ↓ in AS group (p=.003). TD group ↑when eyes are cut into halves (p<.001). No difference when eyes intact. **No. of fixations**- Puzzle piece: ↑on eyes in TD (p=0.004) than other parts of face. No significant difference In mouth area. Recognition photos: ↑ on outer facial features in AS (p<.001). TD had ↑ on eyes (p<.001) and mouth (p=.049) compared to AS. **Fixation duration**- Puzzle pieces: TD ↑ viewing of eyes (p<.001) and mouth (p=0.004). Recognition photos: AS ↑viewing of eyes (p=.001) and mouth (p<.001). | Strong quality (score: 21/24). Reliable and valid instrument used. Appropriate description of sampling strategy and inclusion/exclusion criteria. Blinding of investigators not reported. Possible confounding factor as IQ was not controlled. Possible Type II error calculated (d=0.30). |
| Grelotti et al. [18] , level V | Autism | 2 | N/A 11 Y and 17 Y | 1 | 10 Y | CH & ADO | Static | Familiar/Unfamiliar, objects and cartoons | **fMRI Simple face perception-** *Task 1:* respond true or false to labels. *Task 2:* discriminate whether the two faces are same or different (no delay) | **Reaction time**- TD ↑ to human and Pokemon faces compared to objects or Digimon. DD (autism) ↑ in Digimon than faces or objects but ↓ in faces compared to objects. **fMRI activation**: DD showed activation in fusiform gyrus and amygdala when viewed Digimon faces but not in faces. | Adequate quality (score: 14/22). Reliable and valid instrument used. Individual case study design was used. It would be more appropriate to use a case-control design. Small sample size and baseline characteristics were not controlled. |
| Kleinhans et al. [24], level III | ASD | 19 | 21.9 (5.9) Y | 20 | 24.7 (7.9) Y | ADO | Static | Inversion effect with neutral faces and houses. | **fMRI Simple face perception-** Simultaneous identification (respond when stimulus is identical in succession) | **Recognition accuracy-** No significant difference **Reaction time**- ASD group responded longer. **fMRI activation-** ***Amygdala:*** ↓ in ASD group compared to TD (p<0.05) ***Fusiform:*** no differences | Strong quality (score: 18/22). Reliable and valid instrument used. Sampling method is unclear. Provided insufficient information of inclusion/exclusion criteria of the participants. Small sample size. Possible Type II error calculated (d=0.71). |
| Kylläinen et al. [35] , level III | Autism | 10 | 9.10 (1.5) Y | 10 | 9.1 (1.2) Y | CH | Static | Task 1: direct gaze or eyes closed faces compared with motorbike. Task 2: Averted gaze (left or right) | **MEG. Simple face perception-**simultaneous identification (respond when stimulus is the same) | **Recognition accuracy-** Task 1: TD ↑ in faces than Autism (p=0.005). no differences in object recognition. Task 2: significant results between groups (p=0.026). **MEG-** exhibit similar pattern in both groups. | Strong quality (score: 19/22). Reliable and valid instrument used. Description of sampling was not described. Small sample size. Possible Type II error calculated (d=1.26 is required). |
| McPartland et al. [39] , level III | Autism, AS | 15 | 21.2 (8.3) Y | 21 | 24.6 (6.3) Y | AD | Static | Wechsler | **EEG. Facial memory-** old-new | **Recognition accuracy-** ASD ↓in comparison to TD group (significant p<0.01). **EEG measures- *N170 latency:*** ↑in ASD (p<0.01), TD exhibited longer differences in latencies in inverted conditions compared to upright and ASD group showed minimal differences. | Strong quality (score: 20/22). Reliable and valid instrument used. Sampling strategy and inclusion/exclusion criteria were sufficiently described. Small sample size. |
| McPartland et al. [25], level III | ASD | 36 | 11.2 (3.4) Y | 18 | 12.6 (2.4) Y | CH | Static | Inversion effect- neutral faces and houses | **EEG. Benton Facial Recognition test. Simple face perception-**simultaneous identification (respond when stimuli was repeated) | **Recognition accuracy-** Face recognition ↓ in ASD (p<0.01).**EEG measures-** *P1 amplitude*- no significance, *P1 latency*- no significance, *N170 amplitude*- Faces vs house: Right lateralisation was observed in TD (p<0.01). ASD group showed bilateral activation (p<0.05). Upright Faces vs inverted faces: TD ↑ in inversed condition but ↓ in ASD (p<0.05), *N170 latency-* Faces vs houses: N170 ↑ (p<0.05) in TD. | Strong quality (score: 20/22). Reliable and valid instrument used. Sampling obtained from previous studies. Sample size for the ASD group is sufficient but more participants needed for controls. Possible Type II error calculated (d=0.58). |
| Parish-Morris et al. [36], level III | Non-spectrum, ASD, Autism | 60 | 11.28 (2.89) Y | 50 | 11.34 (3.04) Y | CH & ADO | Dynamic | Averted and direct gaze. Whole faces | **Eye tracking. Simple face perception-**simultaneous by 3AFC | **Recognition accuracy-** TD ↑ recognition than ASD (p=0.008). **Fixation duration**-no significant differences | Strong quality (score: 21/24). Reliable and valid instrument used. Controlled for confounding factors. Sampling strategy was not described. Blinding of investigators was not conducted. Sufficient sample size. |
| Pierce & Redcay [20], level III | Autism, AS, PDD-NOS | 11 | 9.9 Y | 11 | 9.8 Y | CH | Static | Familiar/ unfamiliar faces compared with objects | **fMRI. Simple face perception-**simultaneous identification (respond when stimulus is repeated consecutively) | **Recognition accuracy-** No significant differences. **Reaction time-** no significant differences between ASD and TD but ASD were slower in responding to unfamiliar faces (p<0.05). **fMRI activation-** no significant differences in amygdala or anterior cingulate. ↓ posterior cingulate activity in ASD when viewed familiar faces. ↓fusiform activation when viewing adult unfamiliar face in ASD. | Strong quality (score: 18/22). Reliable and valid instrument used. Sampling strategy and inclusion/exclusion criteria not sufficiently described. Small sample size. Possible Type II error calculated (d=0.03). |
| Reed et al. [26], level III | Autism, AS | 10 | 28 Y | 14 | 34 Y | AD | Static | Inversion effects-face, body postures and houses | **Simple face perception-** slight delay, identify “same” or “different” | **Recognition accuracy-** Similar facial recognition accuracy. | Good quality (score: 17/22). Reliable and valid instrument used. Needed additional information of inclusion/exclusion criteria. Small sample size. Possible Type II error calculated (d=0.02). |
| Kuusikko et al. [19] , level III | Autism, AS | 45 | 11.5 (2.1) Y | TD: 70 P: 73 | TD: 12.4 (2.3) Y. P: 43.1 Y | CH | Static | NEPSY | **Facial memory-** Immediate memory and delayed memory (after 30 minutes): 3AFC | **Recognition accuracy-** facial memory is ↓ in ASD group and ↑ object recognition ability | Strong quality (score: 18/22). Reported possible bias in using the NEPSY assessment as it is normally used for younger children. Thus, reliability and validity may be affected. Sampling obtained from two previous studies. Baseline characteristics were not sufficiently controlled (controls differ in IQ scores). Good sample size. |
| Snow et al. [33] , level III | Autism, AS, PDD-NOS | 22 | 15.96 (2.44) Y | 21 | 16.81 (1.90) Y | ADO | Static | Whole faces and fans | **Eye tracking Simple face perception-** remember 5x6 stimuli, identify 5x12 image old-new | **Recognition accuracy-**ASD group ↓ for faces (p=.001) but not fans (p>0.50). **No. of fixations-** TD ↑ in faces than objects (p<0.0001) but this did not differ in the ASD group. **Fixation duration-** ASD spent less time fixating on the outside primary facial features (p<0.01). | Strong quality (score: 20/24). Reliable and valid instrument used. Sampling strategy was appropriate (controls recruited from the community). Blinding of investigators was not reported. Inadequate sample size. |
| Song et al. [27], level III | Autism, AS | 15 | 9.17 (1.84) Y | 18 | 9.03 (1.27) Y | CH | Static | Part whole effect |  | Both TD and ASD group used information from the eyes | Adequate quality (score: 13/22). Possible bias in sampling strategy due to recruitment of participants from one hospital and school. Insufficient information provided in results and descriptions of analytic methods were not included. Possible type II error (d=0.95 is required) |
| Sterling et al. [28] , level III | Autism, AS, PDD-NOS | 17 | 23.40 (7.19) Y | 18 | 24.24 (6.86) Y | AD | Static | Familiar/ unfamiliar face | **Eye tracking** | **Recognition accuracy-** no significant differences. **No. of fixations**- TD ↑ in unfamiliar faces compared to familiar faces. No differences in ASD. **Fixation duration-** TD ↑ on eye and mouth. **Reaction time-** no significant differences | Strong quality (score: 20/24). Reliable and valid instrument used. Inadequate sample size. Blinding of investigators was not conducted. Sampling methods were not described. Possible Type II error calculated (d=0.28). |
| Tehrani-Doost et al. [21] , level III | Autism, AS | 15 | 12.80 (3.23) Y | 15 | 10.53 (3.04) Y | CH & ADO | Static | Benton | **Facial memory-**Immediate and delayed memory (after one week) | **Recognition accuracy-**no differences in facial recognition. **Reaction time-** no significant differences | Adequate quality (score: 15/22). Possible bias in sampling strategy as ASD group was recruited from one psychiatry clinic. Inadequate sample size. Confounding factor reported as IQ not controlled. Possible Type II error calculated (d=0.55). |
| Trepagnier et al. [22], level III | ASD | 5 | 18.4 Y | 6 | 19.5 Y | AD | Static | Whole faces and objects | **Eye tracking Simple face perception-** indicated whether the image had been seen | **Recognition accuracy-** ASD ↓ in face recognition and vice versa. **No. of fixations**- ASD ↓ on the central of the face compared to TD. | Poor quality (score: 4/24). Poor reliability and validity of eye tracking measurement due to reported miscalibration. Provided insufficient information in results. Inclusion/exclusion criteria of participants. Baseline characteristics (eg. IQ) were not controlled. Small sample size. Possible Type II error calculated (d=1.7 is required). |
| Webb et al. [40] , level III | Autism, AS, PDD-NOS | 29 | 22.4 (6.1) Y | 28 | 24.0 (7.0) Y | AD | Static | Familiar/ unfamiliar faces and houses. Wechsler | **EEG. Facial memory-** immediate and delayed (after 30 minutes) memory. Recognition: presented with 48 stimuli (old-new) | **Recognition accuracy-** ASD ↓in both delayed and immediate memory (p<0.05). **ERP responses**- showed similar P2, N250 and N400. | Strong quality (score: 21/22). Reliable and valid instrument used. Baseline characteristics controlled. High reliability and validity of instruments used. Sampling selection not sufficiently described. |
| Webb et al. [32], level III | Autism, AS, PDD-NOS | 32 | 23.1 (6.9) Y | 32 | 23.7 (6.7) Y | AD | Static | Inversion effect for faces or houses Wechsler Benton | **EEG. Facial memory-** immediate and delayed (after 30 minutes) memory. Recognition: presented with 48 stimuli (old-new) | **Recognition accuracy-** ASD ↓ in both face and object recognition. **ERP responses-** Both groups demonstrated similar P1 (positive)-p <0.001 and N170 (negative) -p <0.001 amplitude to faces. ***P1:*** TD ↑ (p=0.01) P1 amplitude during inverted stimuli than upright stimuli. ***N170:*** no differences in both groups | Strong quality (score: 21/22). Reliable and valid instrument used. Reported possible bias in results due to increasing public awareness of autism. Selection strategy was not sufficiently described. Good sample size and controlled for baseline characteristics. |
| Wilkinson et al. [29] , level III | Autism | C: 18A: 16 | C: 13.1 (2.2)Y A: 27.5 (9.4)Y | C: 13A: 15 | C: 14.3 (1.7) Y A: 26.9 (7.9) Y | CH & ADO | Static | Whole faces | **Simple face perception-** Slight delay and indicated whether the stimulus is old or new | **Recognition accuracy (Memory awareness accuracy)**- In TD children, ↑ (p<0.01). Autism adult group preformed similarly to the TD Adults. | Strong quality (score: 19/22). Stated possible bias in measurements of memory awareness (used a three point scale which may not be sensitive). Inadequate sample size. Type II error calculated (in adult group) (d=0.53). |
| Wilson et al. [34] , level III | Autism, AS | 27 | 10.07 (2.05) Y | TD: 47 AM TD: 27 | TD: 8.52 (2.90) Y. AM TD: 6.96 (1.78) Y | CH & ADO | Static | Whole faces (different races) | **Simple face perception-** simultaneous VPC | **Recognition accuracy-** mixed results. Some displayed similar facial recognition ability in relation to TD while others performed significantly below (p<0.001). | Strong quality (score: 20/22). Instruments tested for reliability and validity. No reports of ceiling/floor effects. Insufficient description of inclusion/exclusion criteria of participants. Partially appropriate sample size. |
| Wilson et al. [37], level III | Autism, AS | 11 | 10.21 (2.00) Y | 11 | 10.54 (2.04) Y | CH & ADO | Static | Direct and averted gaze | **Eye tracking Facial memory-** remember 4x 20 faces, VPC | **Recognition accuracy-** ASD group significantly below zero (p<0.001)- age standardised scores. **Fixation duration-** ASD had ↓ time at core features (p=0.04). | Good quality (score: 17/24). Reliable and valid instrument used. Participants were controlled for baseline characteristics. Inclusion/exclusion criteria of participants and analytic methods not sufficiently described. Small sample size |
| Zurcher et al. [38] , level III | Autism, AS, PDD-NOS | 16 | 23.5 (6.8) Y | 18 | 15.8 (5.3) Y | AD | Static | Thatcher illusion | **fMRI. Fine grained perception-** Indicate the location of the thatcherzied illusion | **Recognition accuracy-** Overall, ↑ TD group (p<0.05) but ↑ in ASD group in inverted mouth thatcherized stimuli. ↑in ASD group when cued to the eyes upright condition (p<0.05). **Reaction time-** ↑ in inverted conditions for ASD group (p<0.01). **Brain activation (fMRI) -** ASD had heightened activation on subcortical areas when cued to the eyes in upright condition. | Strong quality (score: 18/22). Reliable and valid instrument used. Participants were controlled for baseline characteristics, good inclusion and exclusion criteria for ASD group and control. However, the sampling strategy was not stated and has a small sample size. |

**S2 Table.** Data extraction form. AS, Asperger syndrome; AD, adults; ADO, adolescents; AFC, altered forced comparison; AM, Ability matched; ASD, Autism spectrum disorder, Benton, Benton Facial Recognition test; CH, children; d, standardised difference; DD, developmentally delayed; EEG, electroencephalography; fMRI, functional magnetic resonance imaging; M, months; MEG, magnetoencephalography; N, total number of participants; NEPSY, Neuropsychological assessment; P, Parents; PDD-NOS, Pervasive developmental disorder not otherwise specified; SD, Standard deviation; TD, typically developing; VPC, visual paired comparison; Wechsler, Wechsler memory scale (WMS) 3^rd^ edition-faces subtest assessment; Y, years; ↑, increased; ↓, decreased
